# Light modulates *Drosophila* lifespan via perceptual systems independent of circadian rhythms

**DOI:** 10.18632/aging.204472

**Published:** 2023-01-06

**Authors:** Jacob C. Johnson, Allyson S. Munneke, Haley M. Richardson, Christi M. Gendron, Scott D. Pletcher

**Affiliations:** 1 Department of Molecular and Integrative Physiology, University of Michigan, Ann Arbor, MI 48109, USA; 2 Program in Cellular and Molecular Biology, University of Michigan, Ann Arbor, MI 48109, USA; 3 Geriatrics Center, University of Michigan, Ann Arbor, MI 48109, USA

**Keywords:** sensory perception, aging, circadian clock, model systems, shift work

## Abstract

Across taxa, sensory perception modulates aging in response to important ecological cues, including food, sex, and danger. The range of sensory cues involved, and their mechanism of action, are largely unknown. We therefore sought to better understand how one potential cue, that of light, impacts aging in *Drosophila melanogaster*. In accordance with recently published data, we found that flies lived significantly longer in constant darkness. Extended lifespan was not accompanied by behavioral changes that might indirectly slow aging such as activity, feeding, or fecundity, nor were circadian rhythms necessary for the effect. The lifespans of flies lacking eyes or photoreceptor neurons were unaffected by light kept at normal housing conditions, and transgenic activation of these same neurons was sufficient to phenocopy the effects of environmental light on lifespan. The relationship between light and lifespan was not correlated with its intensity, duration, nor the frequency of light-dark transitions. Furthermore, high-intensity light reduced lifespan in eyeless flies, indicating that the effects we observed were largely independent of the known, non-specific damaging effects associated with light. Our results suggest that much like other environmental cues, light may act as a sensory stimulus to modulate aging.

## INTRODUCTION

Sensory perception influences energy homeostasis, tissue physiology, and organismal aging through neuronal circuits that emanate from sensory tissues and interface with deeper regions of the central nervous system [1]. The molecular study of these relationships is often traced back to the work of Apfeld and Kenyon in the nematode, *Caenorhabditis elegans* [2], and in the years since, sensory effects on aging have been observed across the phylogeny of vertebrate and invertebrate animals [3–8]. Several sensory modalities have been implicated in this relationship including smell, taste, sight, and pain [5], and the ecological cues most often involved are those of food, mates, or danger, detection of which is critical to organism fitness [1].

One ecological cue whose effects on aging have yet to be carefully explored is light. Most animals are exposed to light regularly, and its perception influences nearly all aspects of life from foraging to navigation, from reproduction to survival. Depending on ecological context, light may serve as an attractive or repulsive stimulus. For example, long wavelength light is attractive to planarians, while short wavelength light produces a strong photophobic response [9]. The same light cues can also be interpreted differently, particularly across species, which can result in different behavioral outputs: light is often attractive to larval zebrafish yet repulsive to neonatal mice [10, 11]. Additionally, light cues are the most powerful known entrainment stimulus for circadian rhythms, and they work in tandem with the molecular circadian clock to define daily time perception. Much like the cephalic phase response, in which the smell of food prepares the body in anticipation of consumption, circadian time perception directs changes in physiology and behavior in anticipation of night-time or day-time transitions.

Light is known to have both positive and negative effects on organismal physiology, depending largely on the physical parameters of the light exposure. In humans, different light intensities and wavelengths can positively influence depression scores, cognitive performance, mood, and sleep [12, 13]. On the other hand, in *C. elegans*, lifespan is inversely correlated with the time that worms are exposed to visible light, with effects attributed to photooxidative stress [14]. Short-wavelength visible light increases pupal mortality in several insect species including the vinegar fly (*Drosophila melanogaster*), the mosquito (*Culex pipiens molestus*), and the flour beetle (*Tribolium confusum*) [15, 16]. Increased light intensity also reduces adult lifespan and increases markers of neurodegeneration in adult *Drosophila* [17], which can be reduced by increasing dietary protein content [18]. Again, oxidative and other physical stresses are thought to be the cause [14, 19]. Bright light exposure has been shown to induce neurodegeneration and reduce dopamine levels in the mouse and rat *substantia nigra* [20, 21] potentially by oxidation and generation of cytotoxic byproducts [22]. The damaging effects of UV light on many aspects of biology are well known [23, 24]. The ability of light energy to compromise healthy aging through physical damage is therefore generally accepted, although it is important to note that many of these studies used unnaturally bright light or exposed animals to a light intensity or wavelength outside their normal ecological conditions.

It remains largely unknown whether there are subtler effects of light on aging that do not involve cell-autonomous physical damage but are instead modulated non-autonomously by the sensory systems designed to detect it. There are several indications that this may be the case. First, the pattern of light exposure can influence health independent of duration or intensity. Short pulses of dim light effectively entrain circadian systems, and it has been postulated that organismal health and lifespan are enhanced when the oscillation of light stimulus coincides with endogenous circadian periods [25]. As would be predicted if this postulate is correct, shift work in humans is associated with negative physical and mental health effects [26–28], including cancer as well as metabolic, cognitive, and neurodegenerative disorders [29, 30]. Second, certain types of light have been shown to be beneficial in some contexts. Near infrared light was reported to modestly increase lifespan in *Drosophila* [31], and it has been used to treat Alzheimer’s and Parkinson’s disease [32–34]. Enhanced stress resistance and health span can be achieved by neuron-specific overexpression of the major *Drosophila* photoreceptor cryptochrome gene, *cry,* which is involved in resetting circadian rhythms upon sensing light [35, 36]. Third, in the mouse, light perception through photosensitive retinal ganglion cells modulates body temperature and sleep independent of the molecular circadian machinery [37].

We sought to test whether moderate amounts of visible light influence aging in *Drosophila* and, if so, whether such effects involve sensory perception. In accordance with published data, we observed that lifespan was robustly extended in both male and female flies when they were aged in constant darkness (DD) relative to siblings aged in standard conditions where lights oscillated in a 12 hr: 12 hr on:off pattern [17]. Slowed aging was not due to behavioral differences in the dark, such as self-dietary restriction, changes in locomotion, or reduced reproduction. Flies that lacked light-sensitive neurons and molecules failed to exhibit lifespan extension in the dark, and activation of these same neurons in the absence of environmental light reduced lifespan, suggesting that light modulates lifespan, at least in part, by visual perception. Lifespan extension in constant darkness was independent of the pace or amplitude of molecular circadian rhythms and independent of perceived time, as measured by the number of subjective days and nights the flies experienced during their lifetime. These studies suggest that much like food, light may influence aging through direct physical effects on cells as well as through indirect effects utilizing sensory systems designed to adjust behavior and physiology in the expectation of temporal changes in the environment. Elucidating the molecular and neuronal mechanisms underlying these sensory-dependent effects of light on aging is an attractive avenue for identifying novel therapies that promote healthy aging.

## RESULTS

### Constant darkness increases *Drosophila* lifespan independent of key aging-related behaviors

**Figure 1:**
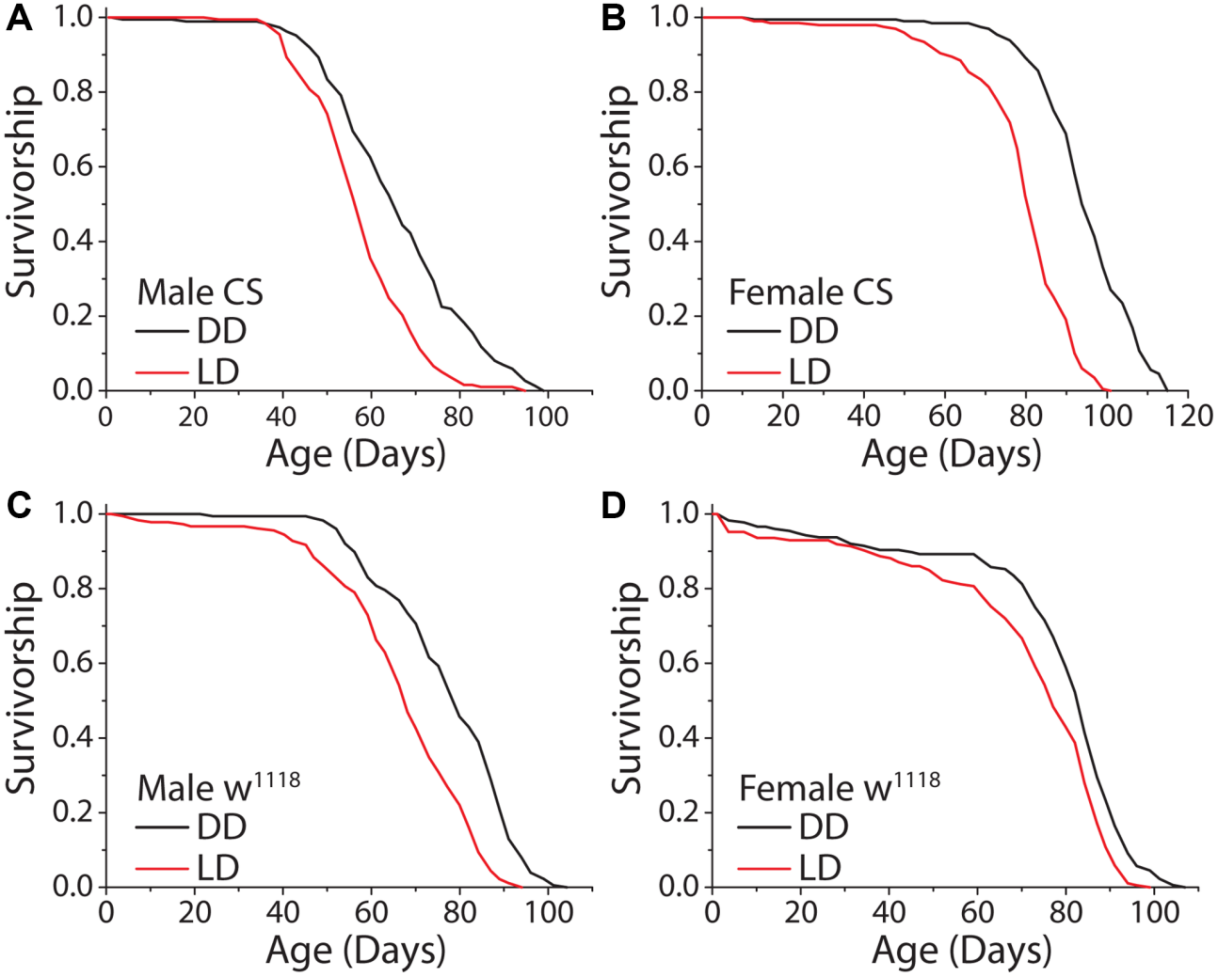
**Constant darkness increases fly lifespan.** (**A**) Removing flies from a standard 12 hr: 12 hr light cycle (LD) and housing in constant darkness (DD) increased fly lifespan in WT Canton-S (CS) male flies (LD *n* = 197, DD *n* = 188; *P* < 0.0001). (**B**) This affect was robust and replicated in female flies (LD *n* = 200, DD *n* = 196; *P* < 0.0001). (**C**, **D**) Light causes significant lifespan shortening in a second laboratory strain *w^1118^*. Both male (**C**) (LD *n* = 181, DD *n* = 171; *P* < 0.0001) and female (**D**) (LD *n* = 186, DD *n* = 176; *P* = 0.00017) flies showed lifespan extension when aged under DD as compared to LD conditions.

We first asked whether the complete loss of a light stimulus modulates lifespan in *Drosophila*. We therefore compared the lifespans of flies aged under constant darkness (DD) with those aged under conventional conditions comprised of repeated 12 hr: 12 hr light:dark (LD) cycles. Preliminary experiments revealed that incubator-to-incubator variability in temperature and humidity, even among units from the same manufacturer programmed to the same conditions, were sufficient to induce significant changes in lifespan. To avoid having such differences confound effects that might be caused by different light regimes, we constructed light compartmentalization structures in a single incubator within which light was maintained and between which temperature was measurably indistinguishable (e.g., over a 60 day period the mean temperature in the dark compartment averaged 25.34°C [SD = 0.35°C] while the temperature in the 12 hr: 12 hr LD cycle compartment was 25.26°C [SD = 0.27°C]). In addition, we used lights with a warm spectral profile similar to indoor lighting commonly used in the home (Supplementary Figure 1). When we aged flies under DD and a standard 12 hr: 12 hr LD cycle, as has since been reported [14, 17], we found that flies of both sexes were significantly longer-lived under constant darkness (Figure 1A, 1B); mean and maximum lifespan was increased up to 19% and 14%, respectively. This effect was consistent across experimental replicates and genetic strains, suggesting that it is robust and not a genotype-specific phenomenon (Figure 1C, 1D).

**Figure 2:**
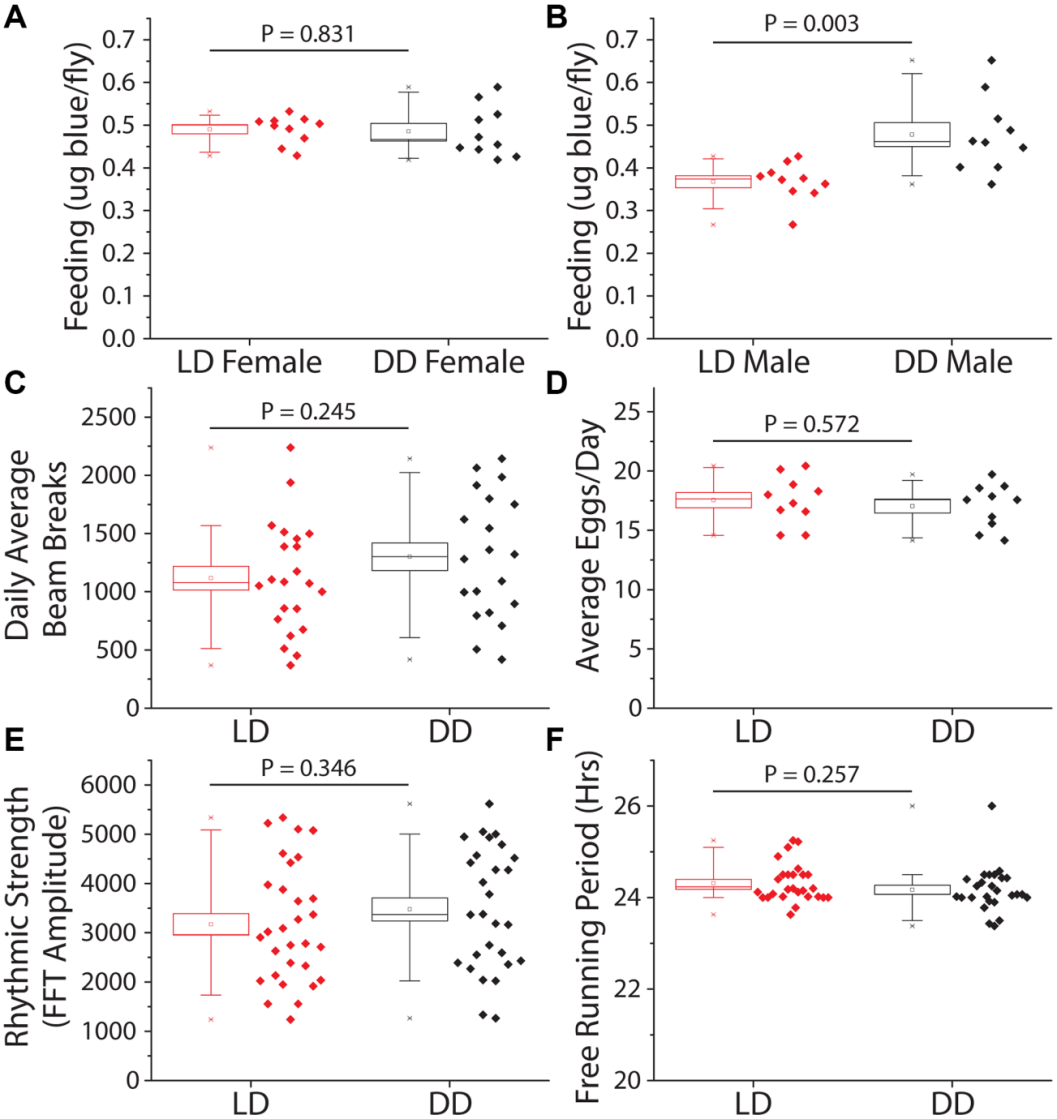
**Constant darkness has no effect on feeding, locomotion, fecundity, or circadian function.** Several aging-related behaviors were measured after 14 days (activity and fecundity) or 21 days (feeding and circadian measures) under LD or DD conditions. (**A**) Female dye labeled food consumption over 24 hours was not significantly different in dark reared flies as measured by dye excretion (*n* = 10 per treatment group of 15 flies each, *P* = 0.83). (**B**) Males reared and kept under dark conditions ate, as measured by dye excretion, significantly more than those under LD conditions (*n* = 10 per treatment group of 15 flies each, *P* = 0.003). (**C**) There was no significant difference in average daily activity as measured by beam breaks in the Trikinetics DAM system over 5 days (LD *n* = 22, DD *n* = 20; *P* = 0.245). (**D**) Number of eggs laid across 7 days was not significantly altered by a 14-day LD cycle when compared to flies reared in DD (*n* = 10 per treatment group of 5 females each; *P* = 0.572). (**E**, **F**) Circadian health was measured by exposing both male LD and DD pretreated flies to a two-day 12 h: 12 hr LD schedule then placing both under free running (DD) conditions to assess rhythmic strength and free running period. Neither rhythmic strength as measured by FFT amplitude (**E**) (LD *n* = 30, DD *n* = 28; *P* = 0.346) or free running period (**F**) as measured by chi-square periodogram (LD *n* = 27, DD *n* = 26; *P* = 0.257) showed an effect of prior light environment.

We next investigated behavioral changes that might indirectly slow aging in constant darkness. First, we measured food consumption to evaluate whether flies were behaviorally limiting their nutrient intake in the dark, thereby executing self-dietary restriction [38, 39]. We observed that total food intake, as measured over a 24 hr period by a modified version of the ConEx feeding assay [40], was not significantly different between female flies previously maintained for 14 days in DD vs. siblings maintained in a standard 12hr:12hr LD cycle (Figure 2A). Interestingly, males subjected to DD consumed modestly but significantly more food than their siblings that were exposed to light (Figure 2B). Second, we measured total activity, which does not directly affect lifespan but may impact caloric balance and long-term health [41]. Flies were first maintained for 14 days in either DD or LD conditions, after which time they were transferred to activity tubes, placed in Trikinetics *Drosophila* Activity Monitors, and measured for five days in their experimental light conditions. We found that flies aged in DD maintained similar overall levels of activity as did flies aged in a 12 hr: 12 hr LD environment (Figure 2C). Third, we examined fecundity as a measure of potential reproductive costs of extended lifespan [42]. Flies aged in DD for 14 days showed no differences in fecundity over a subsequent 7-day period compared to their sibling control flies aged in LD conditions (Figure 2D). Fourth, we asked whether DD affected the decline in circadian rhythms that normally occurs when flies are aged in standard LD conditions [43]. We observed that flies aged in DD for 21 days exhibited measures of rhythm strength and circadian periodicity that were statistically indistinguishable from their siblings maintained in LD conditions (Figure 2E, 2F). We concluded that the extended lifespan observed in flies maintained in DD is not due to diet-restriction, changes in locomotion, reduced reproduction, or improved circadian function.

### Slowed aging in constant darkness is modulated, at least in part, through the perception of light

**Figure 3:**
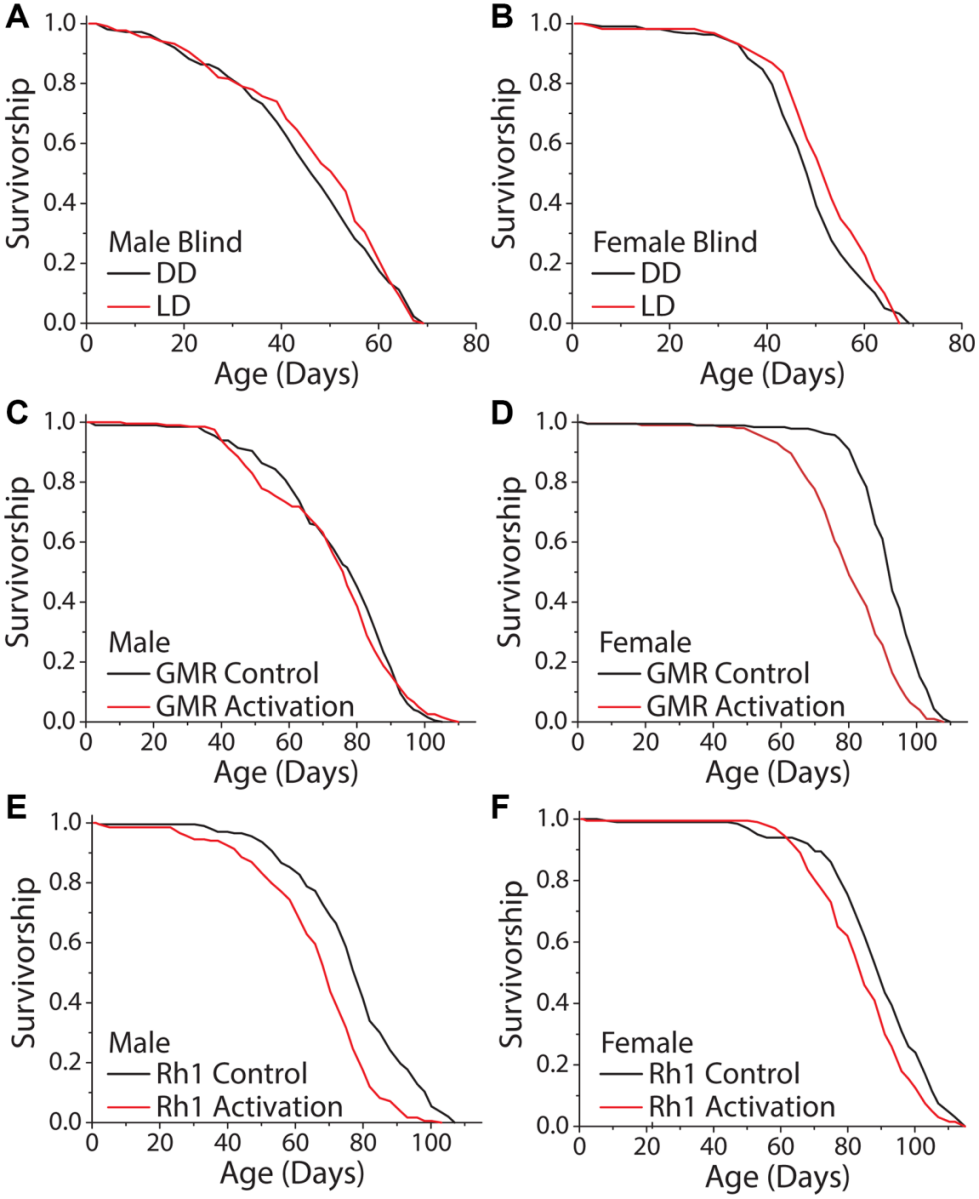
**Activation of visual neurons is necessary and sufficient to mediate the dark lifespan extension.** (**A**, **B**) Flies carrying two copies of the *GMR:hid* transgene, which lack light perception, failed to exhibit an extended lifespan in constant darkness (**A**) males (LD *n* = 213, DD *n* = 223; *P* = 0.288) and (**B**) females (LD *n* = 222, DD *n* = 217; *P* = 0.006). The next 4 panels were conducted in an environment meant to mimic light perception in a standard 24-hour day. Flies carrying a copy of a temperature sensitive cation channel (*UAS-TrpA1*) were used to obtain neuronal activation when at 29°C, and the Gal4 lines were used as background controls. Flies were aged in constant darkness with temperature oscillating 12 hr: 12 hr, 18°C: 29°C. (**C**, **D**) When aged in constant darkness, activation of *GMR*-expressing neurons had no effect in male flies (**C**) (*GMR-Gal4 x w^1118^ n* = 200, *GMR-Gal4 x UAS-TrpA1 n* = 198; *P* = 0.388) but was sufficient to shorten lifespan in females (**D**) (*GMR-Gal4 x w^1118^ n* = 189, *GMR-Gal4 x UAS-TrpA1 n* = 202; *P* < 0.0001). (**E**, **F**) Similarly, spatiotemporal activation of blue light photoreceptor *Rh1* neurons was sufficient to cause a significantly shorter lifespan. This was observed in both male (**E**) (*Rh1-Gal4 x w^1118^ n* = 202, *Rh1-Gal4 x UAS-TrpA1 n* = 203; *P* < 0.0001), and female flies (*Rh1-Gal4 x w^1118^ n* = 200, *Rh1-Gal4 x UAS-TrpA1 n* = 200; *P* = 0.0002).

We next asked whether the perception of light is necessary and/or sufficient to modulate fly lifespan. To determine necessity, we took advantage of visually blind flies that lack eyes and photoreceptor cells. These flies express the proapoptotic gene *hid* under the control of the *Glass Multimer Reporter* (GMR) promoter element, which expresses in the photoreceptor cells and downstream neurons [44]. Flies carrying two copies of the *GMR-hid* transgene are completely eyeless [45, 46]. We found that DD did not increase the lifespans of male or female *GMR-hid* flies compared to siblings aged in standard 12 hr: 12 hr LD conditions (Figure 3A, 3B). To test sufficiency, we sought to mimic light perception while avoiding the potential damaging physical effects of light. We therefore decided to manipulate the activity of light-perceiving neurons and to measure this effect on lifespan in the absence of external light. We again targeted *GMR*-expressing neurons, as well as neurons that specifically express the blue light photoreceptor, *Rh1,* because of the documented effects of this wavelength on lifespan [14, 17]. Spatiotemporal activation was accomplished by employing the *GAL-4/UAS* system to express the temperature sensitive cation *TrpA1* selectively in *GMR* and *Rh1* neurons, respectively. The *Drosophila* TRPA1 channel promotes neuron depolarization only at elevated temperatures (>25°C) and allows for temporal control over cell activation [47]. All experimental flies were aged in DD, and to mimic conventional 12 hr: 12 hr LD conditions, we cycled temperature from 18°C to 29°C on a 12 hr: 12 hr period to activate targeted neurons. Oscillatory activation of all visual neurons with the *GMR* driver and *UAS-TrpA1* proved to have sexual dimorphic effects; male flies were unaffected (Figure 3C) but female flies exhibited a shortened lifespan (Figure 3D). More restricted activation of *Rh1*-expressing neurons reduced lifespan in both males and females (Figure 3E, 3F). These results suggest that light modulates lifespan, at least in part, by visual light perception.

**Figure 4:**
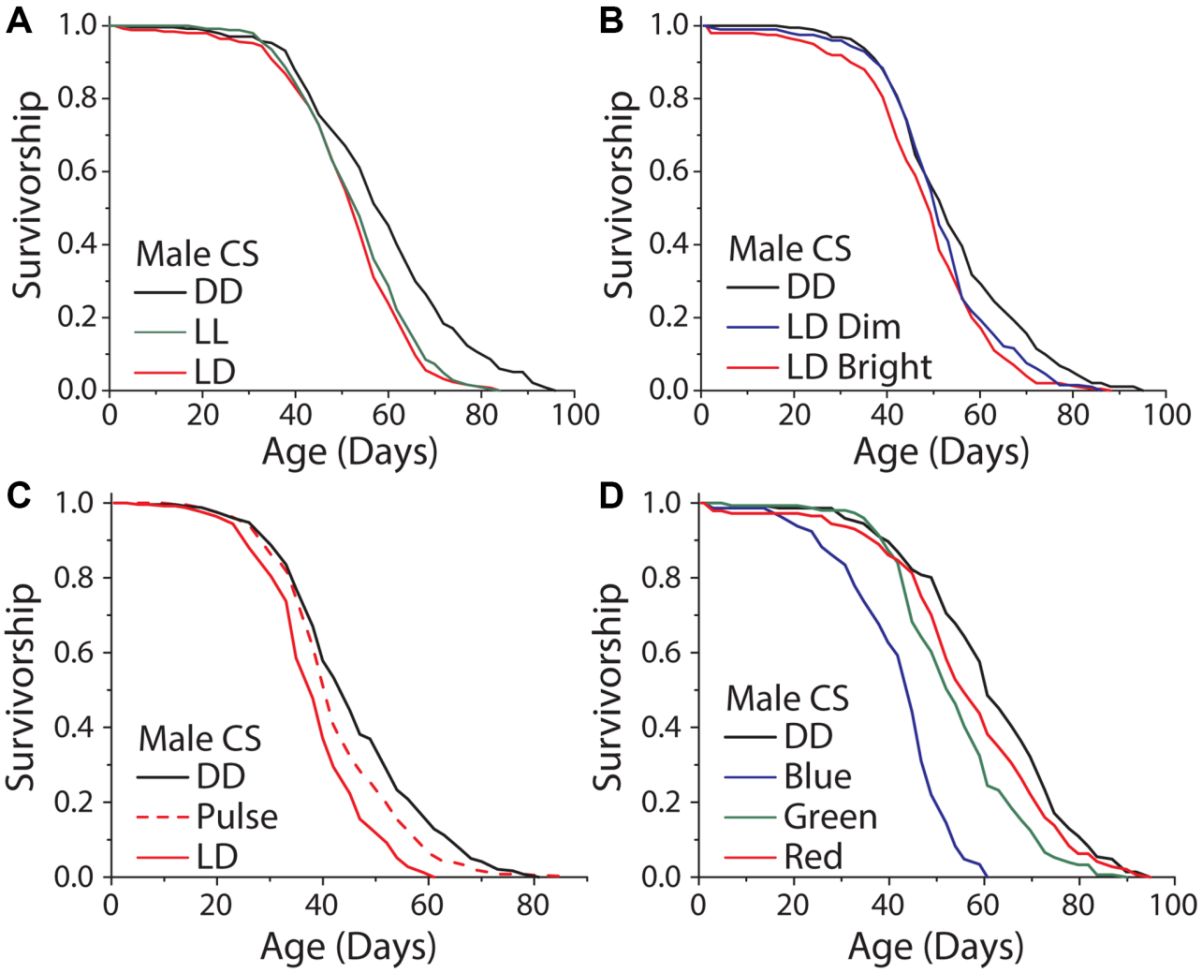
**Light induced damage alone does not account for the dark lifespan extension.** (**A**) Male flies exposed to either 12 hours of light daily or constant light (LL) were significantly shorter lived than those aged under DD (LD *n* = 251, LL *n* = 247, DD *n* = 234; *P* < 0.0001). However, there was no meaningful difference between flies aged under 12 and 24 hours of light (*P* = 0.0304). (**B**) Similarly, there was a significant lifespan shortening effect when flies were aged under 300 and 1050 lux and compared to DD aged flies (300 lux *n* = 198, 1050 lux *n* = 200, DD *n* = 192; *P* = 0.0003). When making pairwise comparisons to DD there was a significant effect of both 1050 lux (*P* < .0001) and a significant effect of 300 lux (*P* = 0.018), however there was no significant difference between the 300 and 1050 lux treatments (*P* = 0.085). (**C**) When exposed to either LD or two, one-hour light pulses a day there was a significant light effect (LD *n* = 251, light pulse *n* = 240, DD *n* = 249; *P* < 0.0001). LD exposed flies were significantly shorter-lived than light pulse exposed flies (*P* < 0.0001) and light pulse exposed flies were significantly shorter lived than DD (*P* < 0.0051). (**D**) When flies were aged under monochromatic light, there was a significant effect of wavelength on lifespan (blue *n* = 145, green *n* = 151, red *n* = 145, DD *n* = 146; *P* < 0.0001). Blue, green, and red light-exposed flies were each significantly shorter-lived than those kept in constant darkness (blue *P* < 0.0001, green *P* < 0.0001, and red *P* = 0.048).

It has been reported that exposure to high amounts of visual light, specifically in the blue range, can directly induce cellular damage and reduce lifespan in the nematode, *Caenorhabditis elegans* [14], and in *Drosophila* [17]. To evaluate whether broad-scale light-induced damage is involved in the lifespan differences that we observed in our standard rearing conditions, we studied the effects of variable exposure time, intensity, light:dark transitions, and wavelength. First, we reasoned that if light energy itself was directly damaging, perhaps by inducing senescence or cell death in visual neurons, then exposure time would be negatively correlated with lifespan. We therefore compared the lifespans of flies aged under constant light (LL) to those aged in 12 hr: 12 hr LD conditions and in DD. While DD reliably extended male lifespan, we found that flies aged in LL were not shorter-lived than those aged in LD conditions (Figure 4A). The same result was observed with female flies (Supplementary Figure 2A). Moreover, we found no significant difference in lifespan between male flies exposed to a 12 hr: 12 hr LD schedule with dim light (300 lux) and those similarly exposed to 5× brighter light (1050 lux; Figure 4B), although in females the dim light treatment had a reduced effect on lifespan compared to bright light (Supplementary Figure 2B).

It is possible that lifespan is subject to threshold modulation in which a small amount of light triggers a maximum effect on lifespan or that transitions from light to dark (and *vice versa*) are important. To test these ideas, we aged flies in conditions where darkness was interrupted by two light pulses each day from 8 am–9 am and 7 pm–8 pm. We chose this design so that the light pulses would coincide with the first and last hours of light under our standard 12 hr: 12 hr LD cycle while also doubling the number of transitions that the flies experienced. Male flies aged in these conditions lived significantly longer than flies exposed to 12 hr: 12 hr LD cycles but shorter than flies aged in DD (Figure 4C). Female flies did not show the same trend (Supplementary Figure 2C). These data suggest that the number of LD transitions is not causal for changes in lifespan and that a straightforward damage model is unlikely to account for our observations. Furthermore, the possibilities remain that light shortens lifespan as a result of perception and/or that the threshold at which light induces damage is above the levels used in these experiments.

Cell autonomous, light-induced damage has been shown to be wavelength dependent. We therefore asked whether different wavelengths of high intensity light were capable of modulating lifespan in our conditions and whether such effects were dependent on perception. Flies exposed to high intensity monochromatic blue (470 nm), green (527 nm), and red (640 nm) light were all significantly shorter lived than flies kept in DD. Shorter wavelengths had larger effect (Figure 4D, Supplementary Figure 2D), which is consistent with previously published studies [14, 17]. Notably, however, eyeless *GMR-hid* flies exhibited a similar response to intense blue light as did control animals suggesting that this treatment influences lifespan independent of visual perception, likely through mechanisms that are distinct from the effects caused by normal levels of visible light (Supplementary Figure 3A–3D). Further, when flies were exposed to the normal levels of broad-spectrum light that were used in LD experiments, or when we activated blue light *Rh1-*neurons for 12 hrs a day, we found there to be no significant changes in stress response gene transcripts (Supplementary Figure 4A, 4B).

### The effects of light perception on lifespan are independent of the molecular circadian clock and of daily time perception

**Figure 5:**
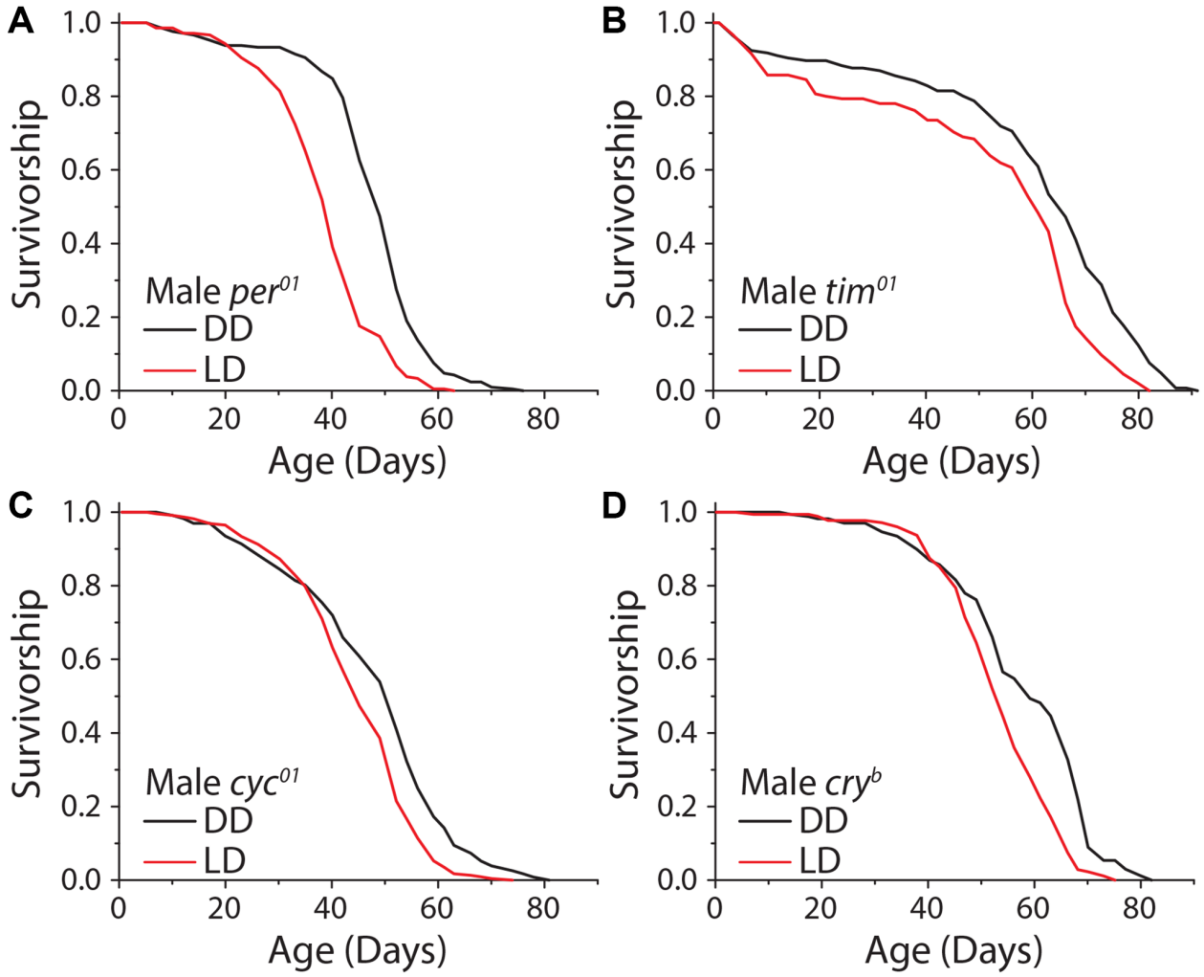
**The molecular circadian clock is dispensable for extended lifespan in DD.** Loss of function mutations in the molecular circadian clock were assessed for their effect on the dark lifespan extension. (**A**) *Per^01^* flies showed a significant lifespan effect when aged under DD conditions (LD *n* = 220, DD *n* = 211; *P* < 0.0001). (**B**) *Tim^01^* mutants were also significantly longer-lived under DD conditions (LD *n* = 155, DD *n* = 146; *P* < 0.0001). (**C**) *Cyc^01^* flies showed a significant lifespan extension when aged under DD conditions as compared to LD (LD *n* = 228, DD *n* = 232; *P* < 0.0001). (**D**) *Cry^b^* flies also showed a lifespan extension when aged in DD conditions (LD *n* = 175, DD *n* = 168; *P* < 0.0001).

Given that sensory perception of light is responsible, at least in part, for extended lifespan in constant darkness, we next asked whether this effect was modulated by mechanisms involved in specifying endogenous circadian rhythms, which are entrained by light patterns. The genes *period (per)* and *timeless (tim)* are essential components of the repressive limb of the molecular clock, and their loss leads to molecular arrhythmicity. Although these mutants are capable of masking, which is showing behavioral rhythms that correspond with light cycles without light anticipatory behavior, and exhibit similar activity patterns as wild-type flies, they will not entrain to the light cycle and are unable to predict the onset of light. We observed that male flies homozygous for a complete loss of function in the *per* allele (*per^01^*) exhibited a significant increase in lifespan under DD, as did male animals carrying a deletion in *tim* (*tim^01^*) (Figure 5A, 5B). To more thoroughly explore whether clock function mediates lifespan extension in the dark, we also tested the potential involvement of the positive limb of the clock by using flies that are mutant for the gene *cycle* (*cyc^01^*), which also results in behaviorally arrhythmic flies. *Cyc^01^* did not abolish the lifespan increase caused by DD in males (Figure 5C). Finally, we tested whether the circadian-light sensor, *cryptochrome (cry),* was required for lifespan extension in constant darkness. Male flies homozygous for the null mutation, *cry^b^*, displayed a significant lifespan extension of similar magnitude to control flies when kept in DD (Figure 5D). While the degree of lifespan changes caused by DD are variable depending on the sex and circadian mutant used, males and females generally show similar trends (Supplementary Figure 5A–5E). These results indicate that lifespan extension in constant darkness is independent of molecular circadian rhythms.

The effects of sensory perception on lifespan are often more pronounced when information provided by sensory systems is uncoupled with the experiences that they were designed to predict [48]. For example, flies that smell food during periods of food scarcity or that detect the opposite sex in the absence of mating opportunities are significantly short-lived [7, 49]. In the context of light and time perception, this situation might be represented by a discordance between the predicted pattern of light cycling provided by the molecular clock and the realization of actual environmental light patterns. Indeed, it is currently thought that such asynchrony reduces lifespan, reproduction, and metabolic health [50–53].

**Figure 6:**
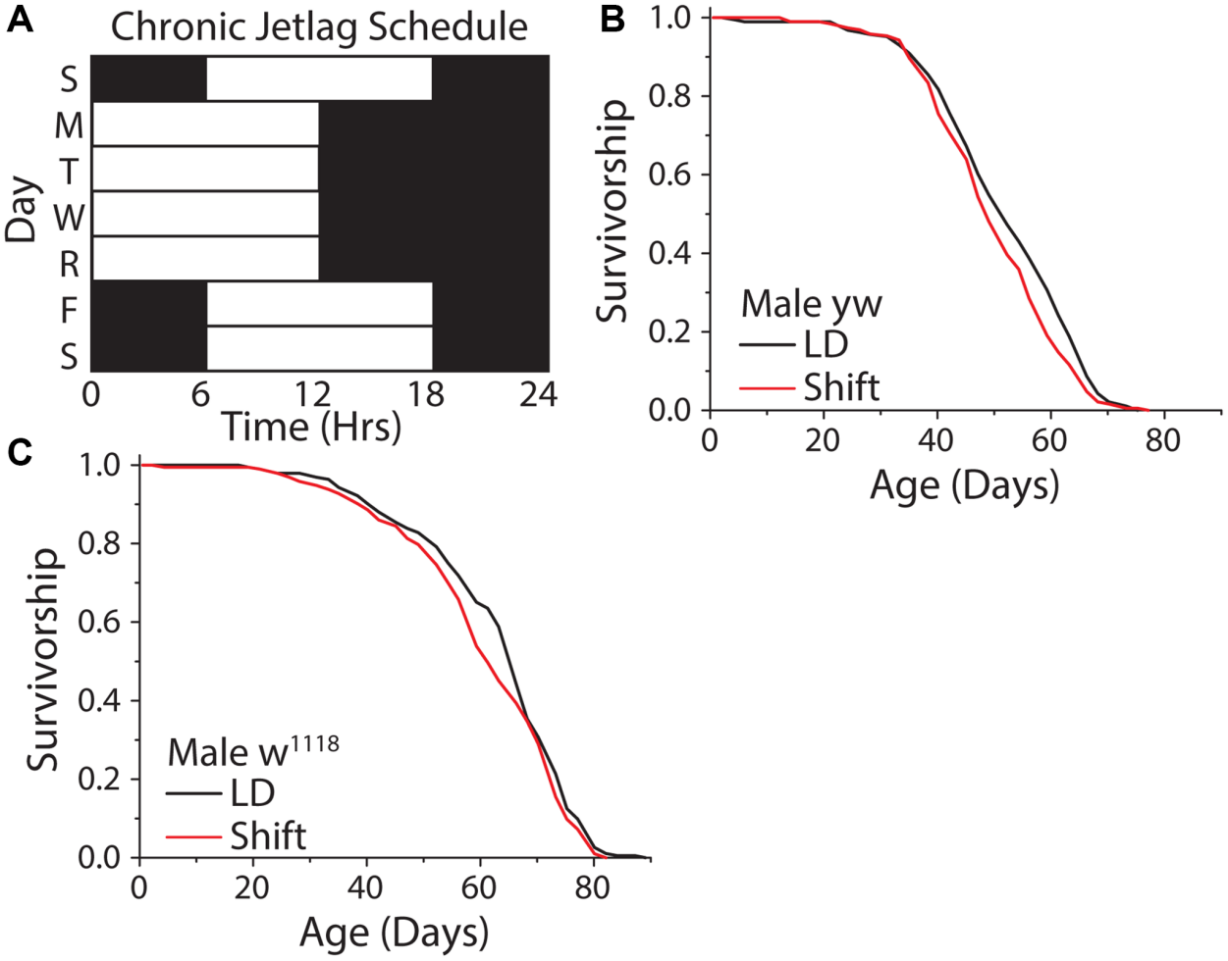
**A weekly 6 hr phase advance and delay had no influence on *Drosophila* lifespan.** (**A**) Experimental design used to subject flies to a light cycle similar in nature to frequent jet lag, or a shift worker who works 4 days a week. Flies were subjected to a six-hour phase advance, then four days later a six-hour phase delay, with individual days always having 12 hr: 12 hr light:dark schedule. (**B**, **C**) The shifting light schedule had little to no effect on male WT lifespan, in both *yellow white* (**B**) (12:12 LD *n* = 188, shift-schedule *n* = 193; *P* = 0.036) and *w^1118^* (**C**) (12:12 LD *n* = 195, shift-schedule *n* = 194; *P* = 0.099) fly strains.

We therefore investigated how different forms of uncoupling between light schedules and the circadian clock impact *Drosophila* lifespan. We began by exploring the effects of repeated exposure to a shifting light cycle (Figure 6A). We chose a light schedule that mimicked human shift workers who travel four days a week or who work nights several days a week and then experience a different schedule on weekends. This was executed by exposing flies to a standard 12 hr: 12 hr light-dark schedule, with lights on from 9 am–9 pm, on Monday-Thursday, imposing a 6-hr phase delay on Friday (i.e., with lights on from 3 pm-3 am), and restoring the normal cycle by applying a 6 hr phase advance on Sunday. A similar schedule had been shown to be detrimental to mouse lifespan [54]. Unexpectedly, this shifting light paradigm had no meaningful effect on fly lifespan, with two different laboratory strains exhibiting a mean reduction in lifespan of ≈3.7% (Figure 6B, 6C).

**Figure 7:**
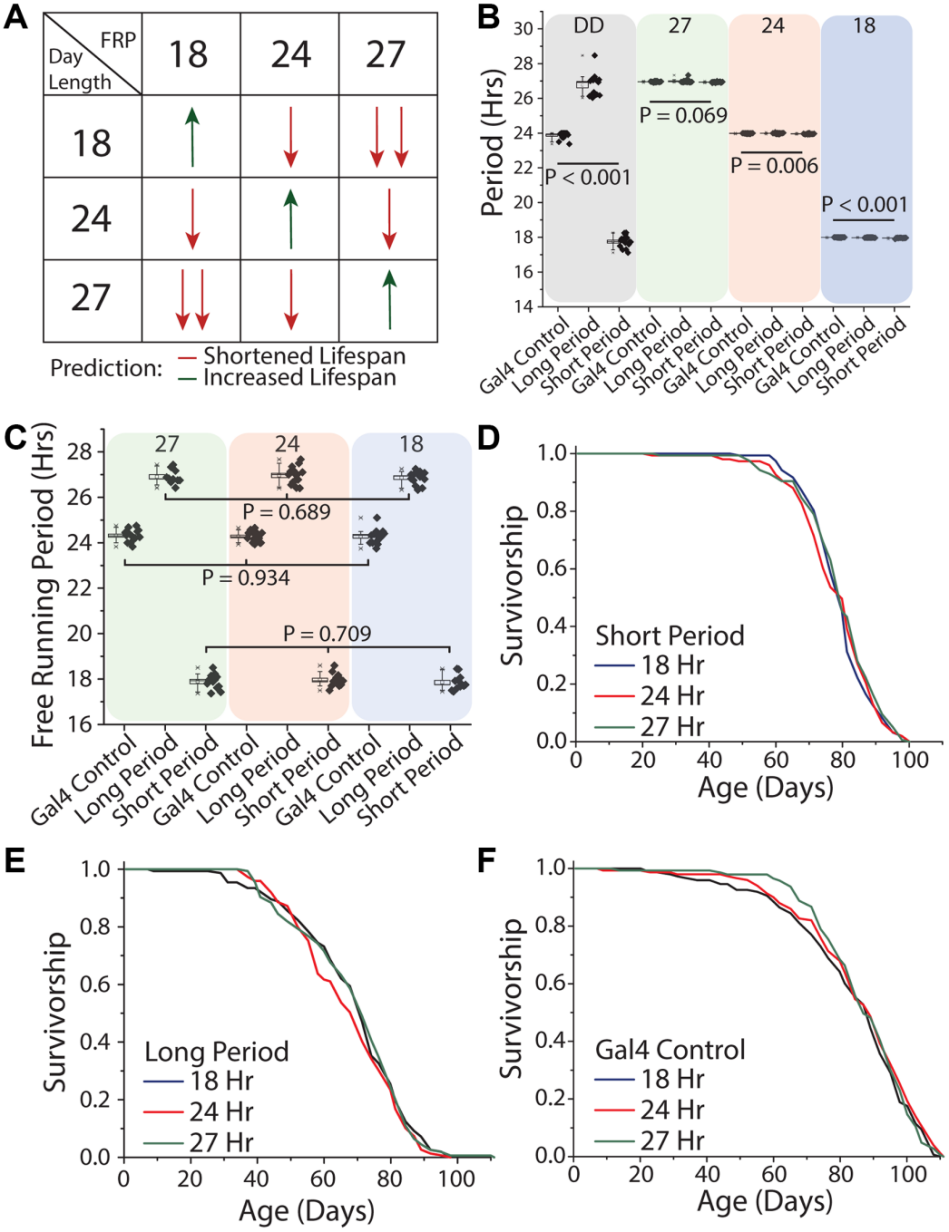
**Uncoupling between light schedules and the circadian clock does not affect *Drosophila* lifespan.** (**A**) Experimental design and lifespan predictions when *Drosophila* with free running periods (FRPs) of 18, 24, and 27 hours were exposed to corresponding light cycles. We predicted as day length further deviates from FRP, that lifespan will be negatively impacted (red arrows) and having a FRP that corresponds with the day length be beneficial to lifespan (green arrows). (**B**) Free running period of 7-day old flies of the genotypes used in the lifespan experiments (grey quadrant), and activity period when exposed to the three light cycles used (green, red and blue quadrants). *Clk856-Gal4 x UAS-DBT^S^* (short day) exhibited a mean period length of 17.8 hours (SD = 0.34) and *Clk856-Gal4xUAS-DBT^L^* (long day) showed a mean period length of 26.8 hours (SD = 0.61). Normal-day *CLK856-GAL4 x w^1118^* (normal day) had a period length of 23.9 hours (SD = 0.19). When exposed to environmental light all flies had an activity rhythm corresponding with the photoperiod. Light cycle day length during recording period is denoted at the top of each colored box. (**C**) After three weeks under light cycles all genotypes were placed in free running conditions and all genotypes reverted to their endogenous free running period. When comparing within a genotype there were no effects of rearing photoperiod, *Clk856-Gal4 x w^1118^* (27 hr *n* = 15, 24 hr *n* = 16, 18 hr *n* = 15; *P* = 0.934), *Clk856-Gal4 x UAS-DBT^L^* (27 hr *n* = 10, 24 hr *n* = 15, 18 hr *n* = 14; *P* = 0.689), and *Clk856-Gal4 x UAS-DBT^S^* (27 hr *n* = 12, 24 hr *n* = 13, 18 hr *n* = 12; *P* = 0.709). Previous light cycle day length is denoted at the top of each colored box. (**D**–**F**) Lifespan of *Clk856-Gal4 x UAS-DBT^S^, Clk856-Gal4 x UAS-DBT^L^*, and *Clk856-Gal4 x w^1118^* was not influenced by environmental light cycle, with all genotypes showing no significant effect of light cycle on lifespan (**D**) short (18 hr *n* = 157, 24 hr *n* = 151, 27 hr *n* = 157; *P* = 0.615), (**E**) long (18 hr *n* = 153, 24 hr *n* = 149, 27 hr *n* = 155; *P* = 0.407), and (**F**) Gal4 control (18 hr *n* = 148, 24 hr *n* = 150, 27 hr *n* = 143; *P* = 0.554).

We next tested different patterns of light oscillation, including oscillation rates that were equivalent to the flies' free running period as well as those that exhibited different degrees of discordance. To do this we expressed mutant variants of the *doubletime* kinase, which is responsible for the phosphorylation of PER and thus the amount of time it takes for the molecular clock to cycle [17, 55]. In this way, we created flies with endogenous periods of 18, 24, and 27 hours, which we refer to as short-day, normal-day, or long-day flies, respectively. Short-day flies expressed *UAS-doubletime-short* (*UAS-DBT^S^*) in clock neurons (using *Clk856-GAL4*), which are the master circadian neurons whose output serves to synchronize all body clocks. Long-day flies expressed *UAS-doubletime-long* (*UAS-DBT^L^*) in those same cells [56], and flies carrying *Clk856-GAL4* but no UAS element served as the control. Flies from each free-running period were exposed to each of three different environmental light:dark conditions of 9 hr: 9 hr, 12 hr: 12 hr, and 13.5 hr: 13.5 hr hours in a factorial design (Figure 7A). This design was chosen to allow direct, within-strain comparisons among treatments in which one environmental light cycle was in line with its endogenous free running period (termed the control environmental condition for that genotype), and two environmental light cycles that were distinct from it. Our design also allowed us to determine whether the magnitude of the difference between environmental and endogenous periods correlate with lifespan effects (Figure 7A).

Before executing the lifespan experiments, we sought to establish that our genetic manipulations were effective in maintaining distinct free-running periods throughout lifespan and that our disparate environmental light cycles were effective in masking them. We found that short-day flies exhibited a mean period length when young of 17.8 hours (SD = 0.34) and that long-day flies showed a mean period length of 26.8 hours (SD = 0.61). As expected, normal-day *Clk856-GAL4* animals, which did not express either altered version of DBT, exhibited a mean period of 23.9 hr (SD = 0.19; Figure 7B). We also found that each genotype effectively entrained to each of the different light cycles and had an activity period that was within 0.1 hr of the diurnal cycle (Figure 7B), even the most disparate. Short-day flies, for example, exhibited 27-hour behavioral rhythms (mean = 26.93 hr, SD = 0.03) when exposed to the 13.5:13.5 hr light:dark regime, and long-day flies expressed an 18 hour behavioral rhythm rhythms (mean = 17.98 hr, SD = 0.02) when exposed to the 9:9 hr regime. When transferred to constant darkness after aging for three weeks in each light environment, flies reverted to their expected genotype-specific free-running periods (Figure 7C), establishing that endogenous rhythms were retained until older ages independent of light condition and that extended durations at different periods did not differentially affect rhythmicity.

We then measured lifespan of each of the three genotypes in each of the three light conditions. We observed that short-day flies exhibited statistically indistinguishable lifespans in conditions of 9 hr: 9 hr, 12 hr: 12 hr, and 13.5 hr: 13.5 hr light:dark regimes (Figure 7D). Similar results were obtained for both long- and normal-day flies: neither genotype exhibited differences in lifespans when aged across the three light regimes (Figure 7E, 7F). In other words, neither the magnitude nor direction of misalignment between oscillations of environmental light and of the endogenous clock affected lifespan in our experiments.

**Figure 8:**
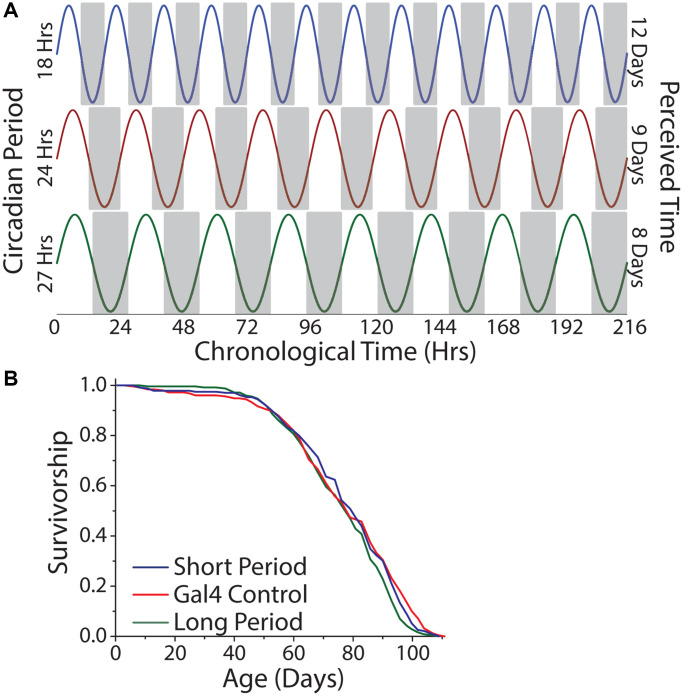
**Lifespan is independent of the number of subjective days lived.** (**A**) Relationship between chronological time and perceived days for short-, normal-, and long-day flies. (**B**) Free running period had minimal effect on lifespan. Animals were aged under free running conditions and a comparison across genotypes was made (short period *n* = 237, long period *n* = 252, Gal4 control *n* = 245; *P* = 0.022).

To examine the hypothesis that perception of time, *per se*, modulates lifespan, we aged the short-, normal-, and long-day flies in constant darkness, which, for a given amount of chronological time, would result in each genotype experiencing a different number of subjective days (Figure 8A). We reasoned that short-day flies, with their 18 hr period, might therefore perceive a more rapid passage of time than would normal-day or long-day flies with their 24 hr and 27 hr periods, respectively, and that a comparison among them would not be confounded by entrainment. We observed that short-, normal-, and long-day flies exhibited similar lifespans in constant darkness (Figure 8B).

When taken altogether, our results from manipulations that were designed to mimic shift work, to study the effects of concordance between endogenous and environmental rhythms, and to examine the effects of perceived time all indicate that the extension of lifespan observed in constant darkness is independent of the molecular clock, that the relationship between circadian timekeeping and external light has little effect on patterns of fly aging, and that the length of life is independent of the number of subjective days.

## DISCUSSION

Similar to previous publications and with a high degree of experimental control, we observed that flies aged under constant darkness lived longer than those aged under typical laboratory conditions (i.e., a 12 hr: 12 hr light:dark cycle). This effect was independent of behavioral changes often associated with lifespan, including feeding, activity, and fecundity, though it should be mentioned these behavioral phenotypes were measured early in life, perhaps before aging-related changes have occurred. Interestingly, blind flies did not show a darkness-mediated lifespan extension, and activation of photoreceptor neurons was sufficient to shorten lifespan and phenocopy the effects of light when flies were kept in constant darkness. These results indicate that there is a perceptual component to the ability of light to modulate aging that was independent of circadian rhythms. Further, when we uncoupled the molecular circadian clock from the environmental light:dark cycles, we found *Drosophila* to be resilient to circadian perturbation. Neither shifting the light cycle nor changing the period of the molecular clock had a meaningful effect on lifespan determination.

We present several lines of evidence indicating that light-induced effects on lifespan are not caused by cell autonomous damage alone. First, the effects of light on lifespan did not scale with exposure time or intensity. Doubling light exposure time had no further effect on lifespan and increasing light levels five-fold, from 300 lux to 1500 lux, only had a modest effect. It should be noted that these light levels are below those of standard *Drosophila* incubators, which are usually measured around 2000 lux, so any potential damaging effects would be less than one might expect in a typical laboratory setting. Second, light:dark transitions, where flies are subjected to repeated startle responses, do not appear to be damaging. Flies exposed to twice-daily light pulses, which are startled twice as often, live longer when compared to those kept under a standard 12: 12 hr light cycle. Third, there were no significant changes in stress response gene transcript levels. Fourth, the effects of our light regime required visual perception. When light perception was muted through genetic ablation of the eyes and photoreceptors, the effect of light on lifespan was lost. However, at high intensities of blue light, ablation of the eyes and photoreceptors was not sufficient to rescue lifespan.

Based on these data, together with published studies, we conclude light is capable of modulating lifespan in *Drosophila* through sensory systems designed to detect it and that at excessively high intensities, particularly of shorter wave lengths, physical damage is pervasive and effects on lifespan are largely independent of sensory perception.

Our work supports the hypothesis that light is similar to food, which has both a direct effect (through cell autonomous nutrient signaling pathways) and an indirect effect (through sensory perception) on lifespan [1]. Much like food/nutrients, where perception of high calorie food acts through odorant receptor activation and results in shorter-lived animals, we predicted that activation of photoreceptor neurons would reduce lifespan [7], which is what we observed. Furthermore, activation of only blue photoreceptors was sufficient to shorten lifespan. Costs of light exposure in our experiments therefore result, at least in part, from the sensing or interpretation of the light cues themselves. These results taken together suggest a biological cost of light perception that is independent of the circadian system and light-induced damage. Further dissection of the molecular and neural mechanisms of light perception may reveal specific photoreceptors and optical processing centers that are required for modulation of lifespan.

Some of our results are inconsistent with previous studies that showed significant effects on lifespan when flies were aged in conditions where external light cycles were discordant with endogenous rhythms [50, 52]. The circadian resonance hypothesis, which states animals’ health and lifespan will be impacted if endogenous period is not synchronized with the environmental period, has been influential, although effects on aging *per se* have not often been examined [17, 57]. Contrary to the predictions of this hypothesis, we found creating discordance between circadian inputs and the internal clock through shifting the flies’ light environment or modulating endogenous period had no meaningful effect on lifespan. These discrepancies may be due to one or more factors. First, to our knowledge, previous studies were performed in multiple incubators with different light environments [50, 52]. We found that environmental variables known to influence lifespan, such as temperature and humidity, were highly variable across different incubators, even when they were programmed to hold identical conditions. Our studies maintained precise control over such factors. Second, it is possible that the circadian resonance hypothesis is more relevant in conditions where there are concurrent stressors – such as mating and predation. Under these conditions, animals must anticipate feeding times of predators and times when mates are most receptive in order to maximize fitness. Similarly, our experiments measured only lifespan, not inclusive fitness, which might be examined by allowing flies of different free running periods to compete. Indeed, it was recently demonstrated in *Drosophila* that measures of fertility and offspring survival were maximized in competitive conditions when the free running period was matched with environmental day length [53]. Lastly, it remains plausible that the circadian resonance hypothesis may be incorrect, and lifespan and health are unaffected by discrepancies between endogenous and environmental periods.

To our knowledge, this is the first study in which *Drosophila* lifespan has been measured while manipulating both circadian period and environmental day length in concordance. Our experimental design provided us with the ability to investigate whether the number of subjective days, and thus a form of perceived time, affects the rate of *Drosophila* aging. We found no relationship between lifespan and subjective time passed, suggesting that circadian time perception does not influence the rate of aging.

Previous studies in *Drosophila* show the impact of light on longevity can be diet dependent at both larval and adult life stages. Larval survival under light conditions can be improved by increasing dietary protein or supplementing food with palmitic acid or biotin [58, 59]. Further, visible light degrades riboflavin in yeast food media, impacting larval survival [58]. Increasing dietary protein given to adult flies also increases survival under light regimes [18]. While dietary content may influence survival, our work with blind flies demonstrates light perception can have lifespan consequences independent of the light induced changes in nutritional environment.

Overall, our results suggest that, like other sensory systems, light perception through visual photoreceptors deserves attention as an intervention that may modulate healthy aging. The possibility that light, or the perception of light, could be used to improve healthy aging is not unreasonable. Near infrared light may improve cell proliferation in culture, and ATP synthesis [60, 61], and far red therapy may ameliorate symptoms of Parkinson’s and Alzheimer’s as well as improve pain management and flexibility in rheumatoid arthritis [62–64]. It is unknown whether the effects of light on lifespan result from damage to sensory neurons, which may lead to systemic effects that reduce lifespan, or from adaptive responses to light perception *per se*, which may recruit signaling pathways that directly modulate aging. Future research on the relationship between light exposure and aging will benefit from focusing on how light impacts the health of visual neurons and how information about the light environment is transduced from the eyes to impact health and aging.

## MATERIALS AND METHODS

### Fly husbandry

All *D. melanogaster* used in this paper were reared using the same method. Experimental flies were age-synchronized using a 3-step procedure. First, mated females and males were placed on a grape juice agarose plate supplemented with live yeast paste for 18–22 hours. The eggs laid during this period were collected briefly in PBS, distributed in 32 ul aliquots into culture bottles containing a modified Caltech Medium (CT food) [65], and reared at 25°C with a standard 12 hr: 12 hr LD cycle. Second, flies that eclosed within a 24 hr window were collected into bottles and maintained on a 10% sucrose/yeast food (SY10) for 2–3 days at 25°C with a 12 hr: 12 hr LD cycle, unless otherwise noted. After the 2–3 day mating period, flies were sorted under light CO_2_ anesthesia into single-sex groups of 20 or 25, unless noted otherwise, and placed into vials containing SY10 media, which was changed every 2–3 days for the duration of their lifespans.

### Fly strains

Canton-S [64349], *yw* [1495], and *w^1118^* [3605] fly strains were obtained from Bloomington *Drosophila* Stock Center. We thank the Todd Lab at the University of Michigan for providing us with *GMR:Hid, GMR-Gal4*, and *Rh1-Gal4* fly stocks. The Giebultowicz lab at Oregon State University generously shared *per^01^, tim^01^, cyc^01^*, and *cry^B^* mutants with us. All *Drosophila* husbandry and experimentation was performed according to the standards accepted by the field. The Shafer lab was kind to give us *Clk856-Gal4, UAS-DBT^L^,* and *UAS-DBT^S^* lines. We also thank the Garrity lab for providing us with the *UAS-TrpA1* line.

### Media recipes

The modified CT food recipe is as follows: 1 L water, 10 g agar, 6 g cornmeal, 30 g sucrose, 55 g dextrose, 45 g yeast, 15 mL tegosept solution (20% tegosept in 90% ethanol), 3 mL propionic acid. Sugar-yeast 10% (SY10) food recipe is: 1 L water, 20 g agar, 100 g sucrose, 100 g yeast, 15 mL tegosept solution (20% tegosept in 90% ethanol), 3 mL propionic acid, and 4 mL antibiotic supplement (1% tetracycline and 2.5% kanamycin) in water.

### Environmental control

Carefully controlled environmental parameters were key for the success of these experiments. As such, all experiments involving comparisons across environmental light cycles were carried out within the same Percival incubator. Individual light cycles were maintained within 3, light-tight, cabinet drawers installed in the incubator. The lights used for the monochromatic light experiments were LEDs sourced from Luxeon Star which had wavelengths of blue (470 nm), green (527 nm), and red (640 nm). They were run on an automatic 12 v timers to create a LD cycle. The lights used for all white light LD and DD experiments were DIODER LED strip lights (item model #: 201.194.18) with an advertised color temperature of 2700K controlled by a digital timer. To compensate for heat produced by the lights, the dark cabinet drawer also contained the same lights on the same schedule, but they were contained within a light-tight aluminum box. Individual cabinet drawer temperature, humidity, and barometric pressure were determined to be statistically indistinguishable in all 3 boxes. Unless noted, temperature was maintained at 25°C +/−0.5°C, and humidity was maintained at 70% with fluctuations of up to 20%. Light spectral profile was determined with a Sekonic C-800-U spectrometer.

### Lifespan assays

Adult male and female flies (aged 2–3 days post-eclosion) were collected from controlled larval cultures and mating conditions (see above), sorted, and transferred into standard vials (20 or 25 flies per vial) containing SY10 media. The number of flies used per treatment group at the start of the lifespan is given in the figure legends. Actual number used in the analysis is noted in the figure legends. Cohort censuses were taken every 2–3 days, at which time flies were transferred to fresh SY10 media. Experiments were coordinated using DLife computer software [66]. For experiments in which flies were aged under dark conditions, transfers occurred under indirect, dim red light (5 lux).

### Temperature-dependent neuronal manipulations

Temperature was used to activate *GMR* or *RH1* expressing neurons in adult flies. Parental crosses for activation strains were *GMR-Gal4 x UAS-TrpA1* and *Rh1-Gal4 x UAS-TrpA1*, while the control crosses were *GMR-Gal4 x w^1118^* and *Rh1-Gal4 x w^1118^*. The *UAS-TrpA1* line used was backcrossed to the *w^1118^* line used for 10 generations to minimize potential genetic background differences. For all crosses, eggs were collected and raised in 18°C 12 hr: 12 hr LD. This temperature was maintained until the beginning of the experiment to ensure there would be no neuronal activation during development. At the beginning of the lifespan experiments flies were transferred to a Percival incubator in which they were maintained in constant darkness under temperature oscillations of 12 hr: 12 hr, 18°C: 29°C. *TRPA1* activation was designed to mimic daytime light perception, and therefore flies were transferred to new media during the 29°C period.

### Activity measurements

Adult male flies (aged 2–3 days post-eclosion) were collected from controlled larval cultures and mating conditions (see above), sorted, and transferred into standard vials (20 flies per vial) containing SY10 media. They were subsequently maintained in either 12 hr: 12 hr light-dark (control) conditions or in constant darkness for 14 days. Flies were then sorted individually into 5 mm × 65 mm polycarbonate tubes (TriKinetics part # PPT5x65), with the same sugar-yeast media placed at one end of the tube. Activity tubes were then loaded into *Drosophila* Activity Monitors (TriKinetics part # DAM2), and monitors were then transferred back into their respective light condition. Recording began 24 hours after the flies were loaded into the activity tubes to allow for acclimation to experimental housing conditions. Data was collected using TriKinetics DAMfilescan, and they were subsequently summed into 30-minute bins. Flies that died during the recording period were excluded from the analysis.

### Circadian period and rhythm calculation

Rhythm and period experiments were conducted using activity measurements as an output of circadian rhythms. Briefly, adult male flies (aged 2–3 days post-eclosion) were collected from controlled larval cultures and mating conditions (see above), sorted, and transferred into standard vials (20 flies per vial) containing 10% SY10 media. Flies were aged for 21 days under 12 hr: 12 hr light-dark (control) conditions or in constant darkness and then were sorted into 5 mm × 65 mm polycarbonate tubes (TriKinetics part # PPT5x65) with SY10 food at one end of the testing tube before being assigned to the *Drosophila* Activity Monitors (TriKinetics part # DAM2). All monitors were placed in the same incubator where they received two days of 12 hr: 12 hr LD then 7 days of DD. TriKinetics DAMfilescan was used for data processing. Actimetrics ClockLab Analysis 3 was used for data analysis. A chi-square periodogram analysis was used to determine period, and fast Fourier transformation analysis was used to calculate rhythmicity values. Flies that died during the recording period were excluded from the analysis.

### Fecundity assay

Adult male and female flies (aged 2–3 days post-eclosion) were collected from controlled larval cultures and mating conditions (see above), sorted, and transferred into standard vials (5 flies of each sex/vial) containing SY10 media. They were subsequently maintained in either 12 hr: 12 hr light-dark (control) conditions or in constant darkness for 14 days, during which time they were transferred to new SY10 media every 2–3 days. After this 14-day acclimation period, fecundity was measured by maintaining each group of flies in their corresponding light/dark conditions and transferring flies to new media daily, after which the number of eggs laid in each vial was recorded.

### Feeding assay

To determine the effects of visible light on feeding we performed a modified version of the ConEx blue feeding assay where only excreted blue is measured [40]. Adult male and female flies (aged 2–3 days post-eclosion) were collected from controlled larval cultures and mating conditions (see above), sorted, and transferred into standard vials (15 flies of each sex/vial) containing SY10 media. Flies were aged 21 days in their respective light environments. Afterward, flies were transferred to new empty vials, which were topped with plastic caps containing SY10 media with 1% FD&C Blue #1. Flies were allowed to feed on dyed food for 24 hours, after which they were frozen for subsequent analysis (see also, ref [40]). Briefly, flies were removed from each vial and the food cap was discarded. Milli-Q water (3 ml) was added to each vial, after which vials were covered (using parafilm) and vortexed. From this 200 uL of solution was transferred into a flat bottom 96 well plate, and absorbance at 630 nm was determined for each well using a BioTek Synergy 2 microplate reader and Gen5 software. Absorbance values were converted to micrograms of food consumed per fly by interpolating a standard curve.

### Quantitative PCR

To determine if the effects of visible or perceived light induce oxidative or stress response genes we assayed a small panel of stress resistance genes in heads of wild type *Canton-S* flies that had been exposed to LD and DD conditions, and *Rh1-Gal4 x UAS-TrpA1* and control *Rh1-Gal4 x w^1118^* flies that were aged under constant darkness under temperature oscillations of 12 hr: 12 hr, 18°C: 29°C. Both experiments were allowed to age for 6 weeks at which point flies were quickly frozen in liquid nitrogen and heads and bodies separated. RNA was extracted using Trizol (Invitrogen) from 3 samples per treatment, with each sample containing 45–50 fly heads. cDNA was synthesized using Superscript III first strand synthesis kit (Invitrogen). Quantitative real-time PCR was performed with Power SYBR green (ThermoFisher). All expression was normalized to the housekeeping gene rp49, and all reactions were performed in triplicate for technical replication.

The following primers were used:

Rp49 F: ATCGGTTACGGATCGAAAA

Rp49 R: GACAATCTCCTTGCGCTTCT

TotA F: GCTTCAGCGTTCCAAAAAGT

TotA R: CTCACGATCTTCGTCGGAAT

Dipt F: ATTGGACTGAATGGAGGATATGG

Dipt R: CGGAAATCTGTAGGTGTAGGT

FMO2 F: CGCAACCAGAAGAAAGCACA

FMO2 R: TGCTCCTGTACGTGTCCAAT

### Statistics

Group and pairwise comparisons on survivorship data were performed using the DLife computer software, and the statistical software R [66]. *P*-values for survivorship data were obtained using log-rank tests. Pairwise comparisons for transcript expression, feeding, fecundity, circadian period, and rhythmicity, and activity data were evaluated using a two-sided independent-samples *t*-test. Group comparisons of circadian period data were evaluated with a one-way ANOVA. All non-lifespan statistical calculations were run in the statistical software OriginPro. For all box plots, box represents Standard Error of the Mean (SEM, centered on the mean), whiskers represent 10%/90%, and the horizontal line represents the median.

### Data availability

All data are available upon request from the corresponding author.

## Supplementary Materials

Supplementary Figures
