# Supplementary material for: Light modulates Drosophila lifespan via perceptual systems independent of circadian rhythms
Source: Aging (Albany NY). 2023 Jan 6;15(2):396–420. doi: 10.18632/aging.204472 (PMC9925688; doi:10.18632/aging.204472)
Supplement: Supplementary Figures [file aging-15-204472-s001.pdf]

SUPPLEMENTARY FIGURES

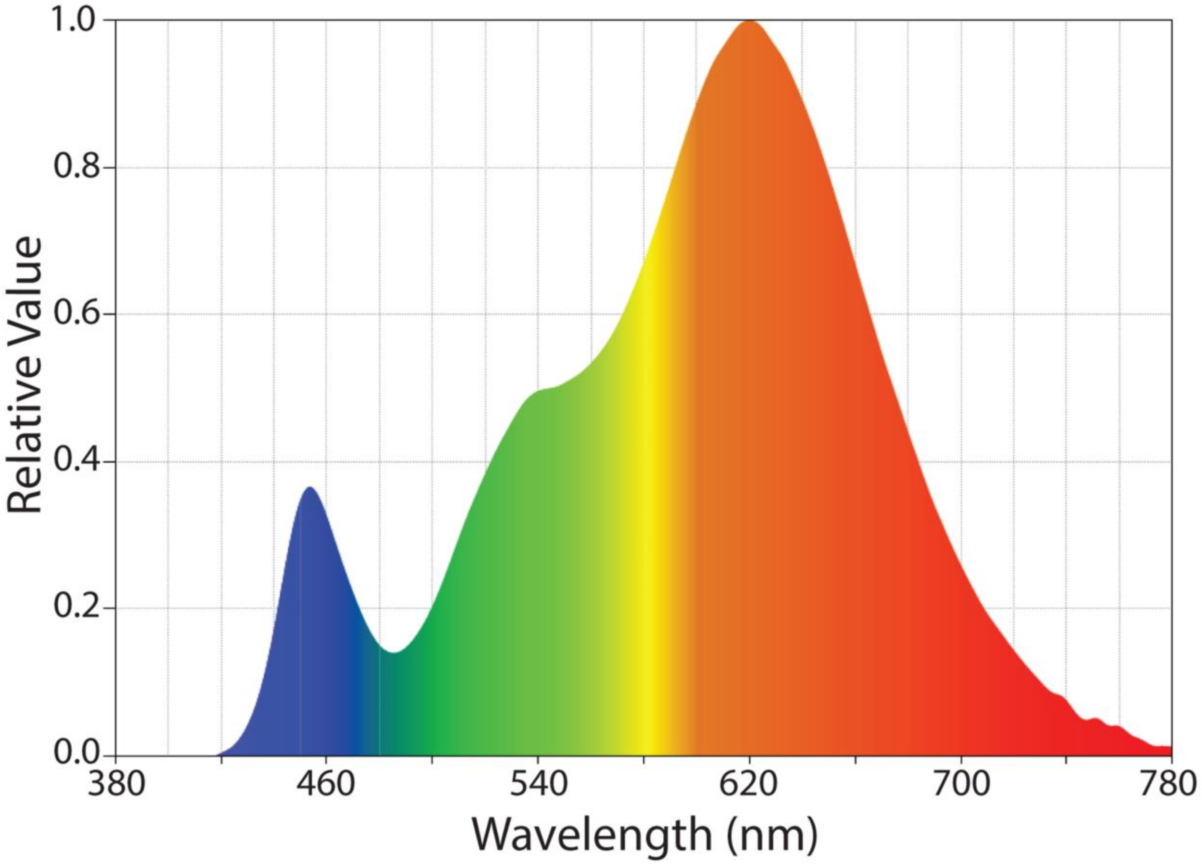

Supplementary Figure 1. Spectral profile of the white LEDs used. Spectral profile of the DIODER LED strips used in LD experiments was determined using a Sekonic C-800-U spectrometer. Values are normalized to maximum value at 640 nm. The profile shown is replicated from the image produced by the spectrometer.

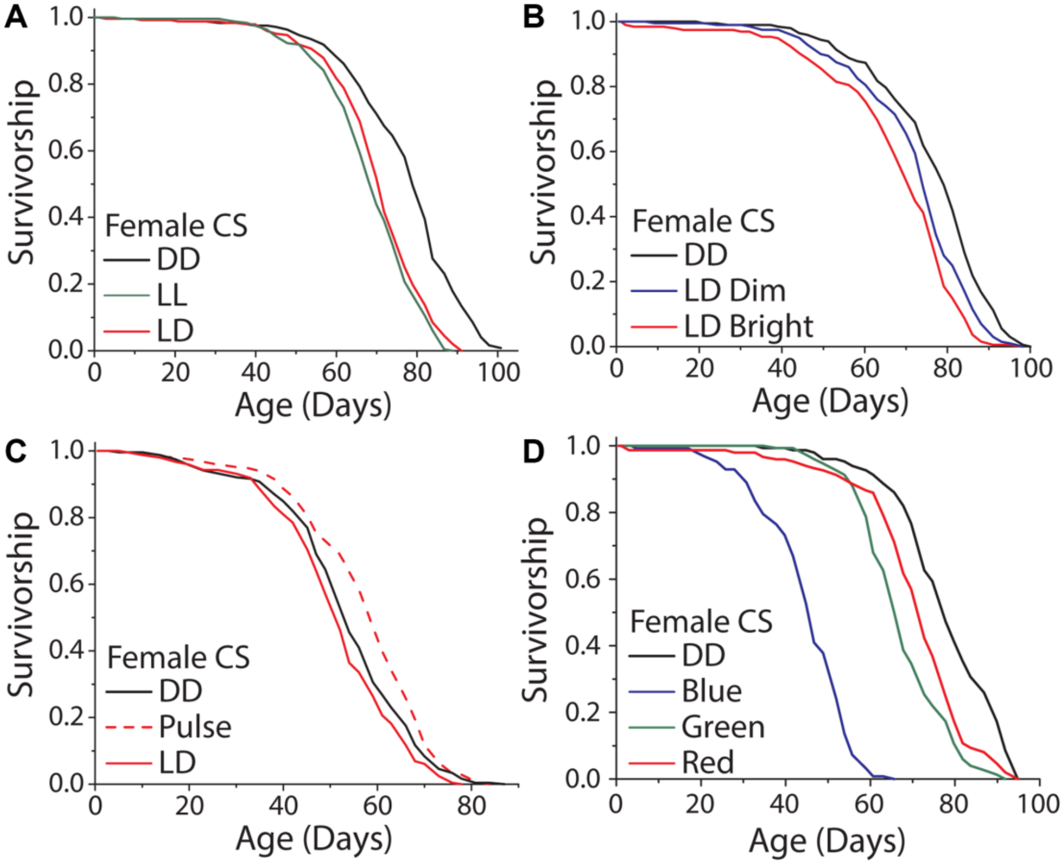

Supplementary Figure 2. Light-induced damage alone does not account for DD lifespan extension. (**A**) Female flies exposed to either 12 hrs of light daily or constant light (LL) were significantly shorter lived than those aged under DD (LD *n* = 247, LL *n* = 246, DD *n* = 246; *P* < 0.0001). However, there was a small but significant difference between female flies aged under 12 and 24 hrs of light (*P* = 0.0467). (**B**) Similarly, there was a significant lifespan effect when female flies were aged under 300 and 1050 lux or DD (300 lux *n* = 200, 1050 lux *n* = 195, DD *n* = 197; *P* < 0.0001). (**C**) When exposed to either 12 hr:12 hr LD or two, one-hour light pulses each day, there was a significant light effect (LD *n* = 247, light pulse *n* = 248, DD *n* = 239; *P* < 0.0001); in females the light pulse exposed flies were longer lived (*P* = 0.003). LD exposed flies remain significantly shorter lived than DD exposed flies (*P* < 0.0253). (**D**) There was a significant effect of wavelength when flies are aged under monochromatic light (blue *n* = 152, green *n* = 150, red *n* = 149, DD *n* = 150; *P* < 0.0001).

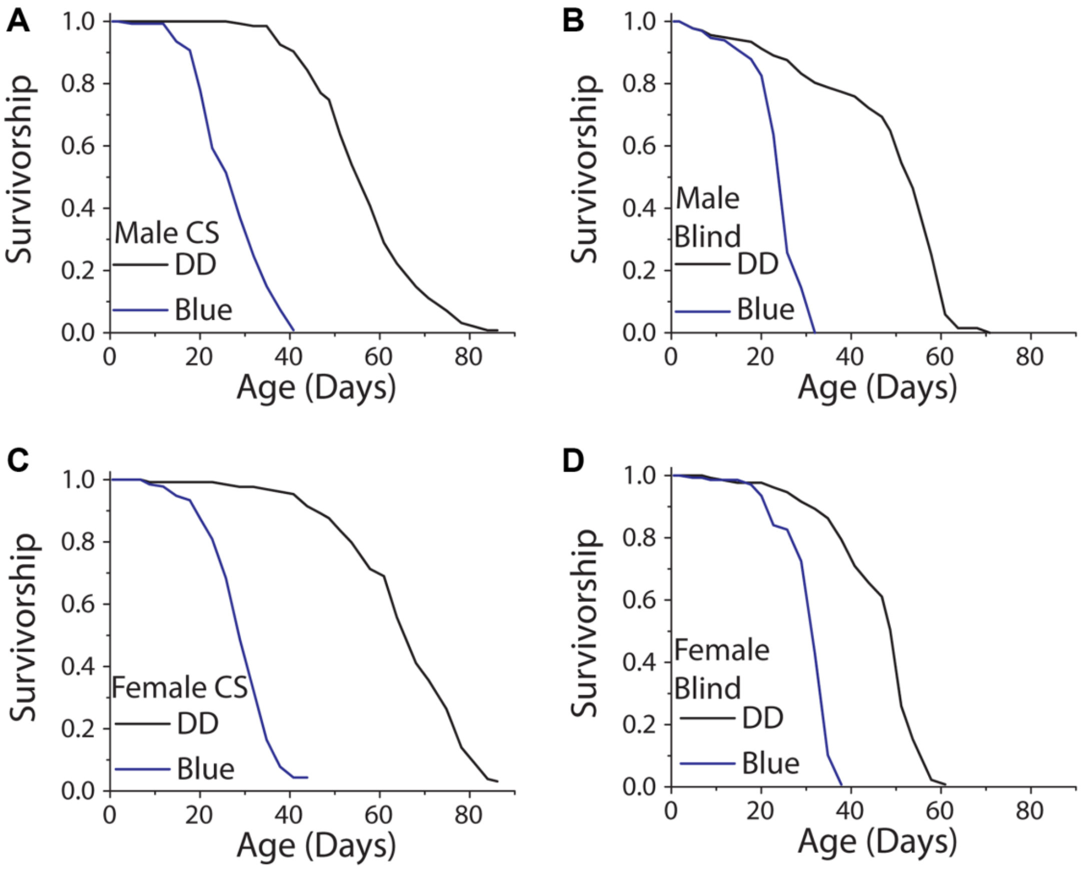

Supplementary Figure 3. Blue light shortens lifespan in sighted and blind *Drosophila.* Flies were exposed to bright monochromatic blue light on a 12 hr: 12 hr LD schedule or kept under dark conditions. (**A**, **B**) Male Canton-S and the blind *GMR:hid* flies lifespan was significantly shortened when aged under monochromatic blue light (CS Blue LD n = 140, CS DD n =135; P < 0.0001); (*GMR:hid* Blue LD n = 135, *GMR:hid* DD n =138; P < 0.0001). (**C**, **D**) Female Canton-S and *GMR:hid* flies also showed significant lifespan shortening when aged under monochromatic blue light (CS Blue LD n = 136, CS DD n =130; P < 0.0001); (*GMR:hid* Blue LD n = 138, *GMR:hid* DD n = 130; P < 0.0001). Plots that end above 0% survival include animals that escaped and were censored prior to the end of the experiment.

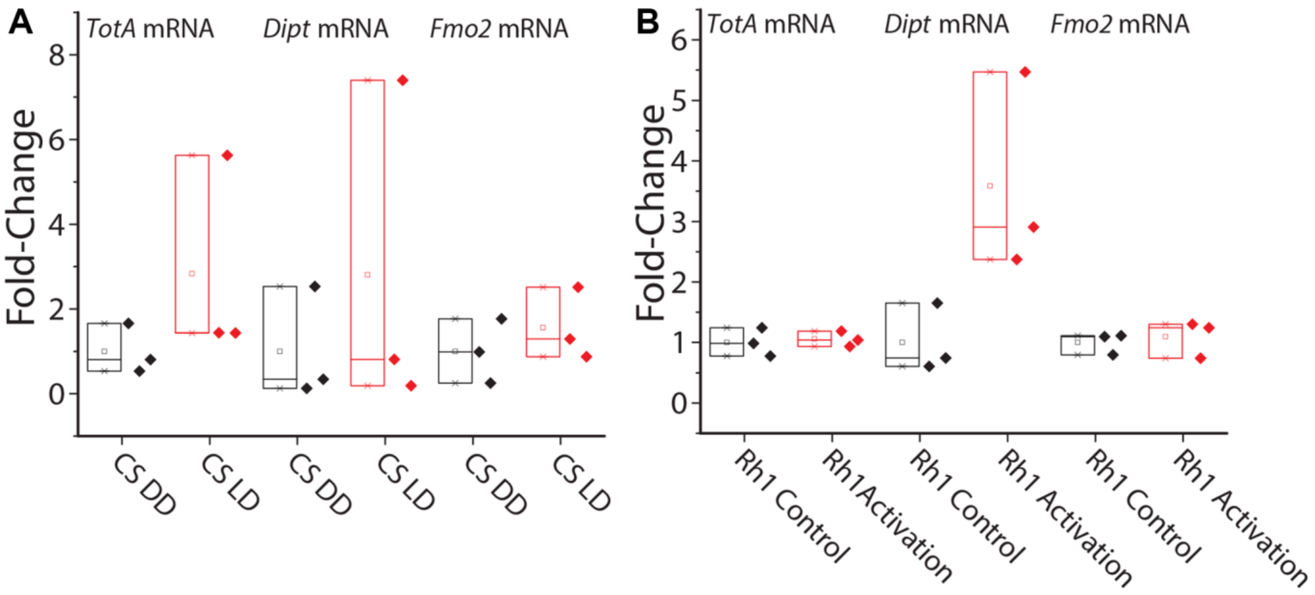

Supplementary Figure 4. Light exposure and blue light sensitive neuronal activation did not increase a subset of stress response gene transcript levels after a 6-week exposure. (**A**) When comparing stress response gene transcript levels in heads from female flies exposed to a 6-week LD regime to those of flies kept under DD conditions we saw no significant change in *Turandot A* (*TotA P* = 0.32), *Diptericin* (*Dipt P* = 0.52), or *Flavin-containing monooxygenase-2* (*Fmo-2 P* = 0.44); (for all genes LD *n* = 3; DD *n* = 3). (**B**) Similarly, when we activated blue light sensitive *Rh1* neurons using a temperature sensitive cation channel (*UAS-TrpA1*) and used the Gal4 line as a background control we saw no significant difference in the same stress response genes for flies that had *Rh1* neurons activated when compared with genetic controls kept at the same activation temperature: *TotA* (*P* = 0.74), *Dipt* (*P* = 0.10), or *Fmo-2* (*P* = 0.67); (for all genes *Rh1-Gal4 x* w^{1118} n = 3; *Rh1-Gal4 x UAS-TrpA1 n* = 3). Flies were aged for 6 weeks in constant darkness with the temperature oscillating 12 hr: 12 hr, 18°C: 29°C.

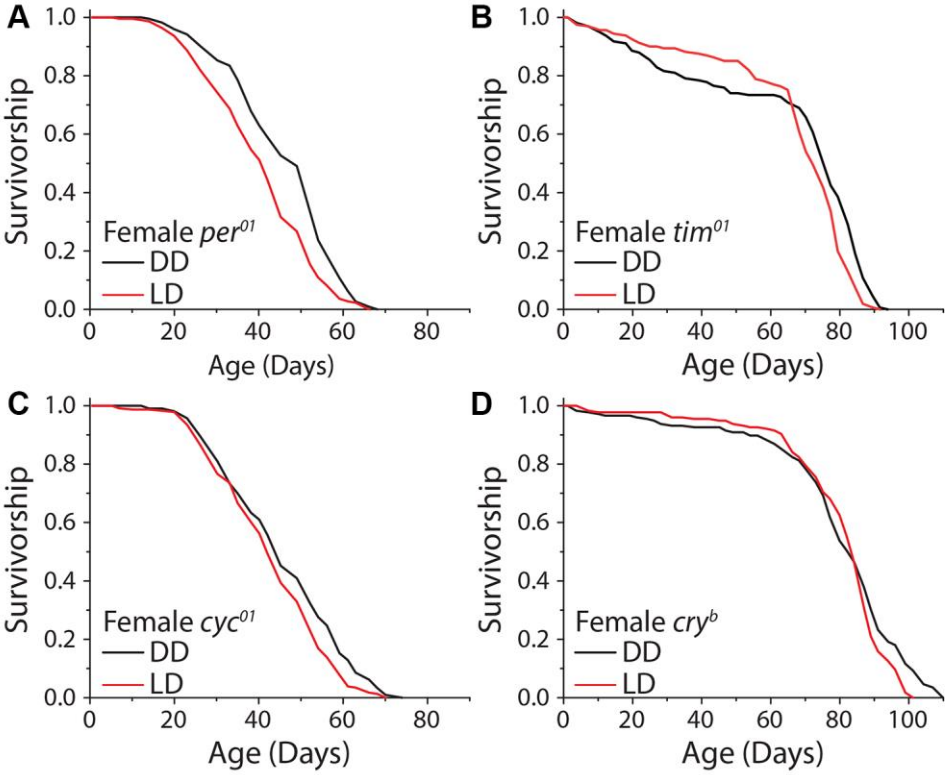

E

Lifespan Summary of Circadian Mutants

Experiment Number	Genotype	Sex	Treatment	Median Age (Days)	P Value (Between LD and DD)
1	per^{01}	Male	LD	40.13	
	per^{01}	Male	DD	49.00	P <0.001
	per^{01}	Female	LD	42.05	
	per^{01}	Female	DD	49.00	P < 0.001
2	per^{01}	Male	LD	59.72	
	per^{01}	Male	DD	63.94	P < 0.001
	per^{01}	Female	LD	67.00	
	per^{01}	Female	DD	74.02	P < 0.001
3	per^{01}	Male	LD	55.14	
	per^{01}	Male	DD	56.89	P < 0.001
	per^{01}	Female	LD	55.14	
	per^{01}	Female	DD	56.89	P = 0.04
1	tim^{01}	Male	LD	61.06	
	tim^{01}	Male	DD	66.25	P < 0.001
	tim^{01}	Female	LD	70.08	
	tim^{01}	Female	DD	75.15	P = 0.001
1	cyc^{01}	Male	LD	45.17	
	cyc^{01}	Male	DD	52.14	P < 0.001
	cyc^{01}	Female	LD	42.05	
	cyc^{01}	Female	DD	45.17	P = 0.005
1	cry^{b}	Male	LD	54.03	
	cry^{b}	Male	DD	59.14	P < 0.001
	cry^{b}	Female	LD	84.21	
	cry^{b}	Female	DD	84.21	P = 0.04

Supplementary Figure 5. In females, components of the molecular clock are not likely required for the dark lifespan extension. Mutations in the molecular circadian clock were assessed for their effect on the dark lifespan extension. (**A**) *Per*^{01} flies showed a significant lifespan effect when aged under DD conditions (LD n = 221, DD n = 224; P < 0.0001). (**B**) *Tim*^{01} mutants are not significantly longer lived under DD conditions (LD n = 161, DD n = 158; P = 0.001). (**C**) *Cyc*^{01} flies showed a small, but still significant lifespan extension when aged under DD conditions as compared to LD (LD n = 231, DD n = 210; P = 0.005). (**D**) *CryB* flies also showed a similar small but significant lifespan extension when aged in DD conditions (LD n = 176, DD n = 176; P = 0.04). (**E**) Table summarizing lifespan data collected using flies with specific mutations in components of the molecular clock.
